# Isolation, characterization and comparative genomics of bacteriophage SfIV: a novel serotype converting phage from *Shigella flexneri*

**DOI:** 10.1186/1471-2164-14-677

**Published:** 2013-10-03

**Authors:** Richa Jakhetia, Kaisar A Talukder, Naresh K Verma

**Affiliations:** 1 Division of Biomedical Science and Biochemistry, Research School of Biology, The Australian National University, Bldg. 134 Linnaeus Way, Canberra ACT 0200, Australia; 2 International Centre for Diarrhoeal Diseases Research, Dhaka, Bangladesh

**Keywords:** *Shigella flexneri*, Bacteriophage, O-antigen modification, Serotype conversion

## Abstract

**Background:**

*Shigella flexneri* is the major cause of shigellosis in the developing countries. The O-antigen component of the lipopolysaccharide is one of the key virulence determinants required for the pathogenesis of *S. flexneri*. The glucosyltransferase and/or acetyltransferase genes responsible for the modification of the O-antigen are encoded by temperate serotype converting bacteriophage present in the *S. flexneri* genome. Several serotype converting phages have previously been isolated and characterized, however, attempts to isolate a serotype converting phage which encodes the modification genes of serotypes 4a strain have not been successful.

**Results:**

In this study, a novel temperate serotype converting bacteriophage SfIV was isolated. Lysogenisation of phage SfIV converted serotype Y strain to serotype 4a. Electron microscopy indicated that SfIV belongs to *Myoviridae* family. The 39,758 bp genome of phage SfIV encompasses 54 open reading frames (*orfs*). Protein level comparison of SfIV with other serotype converting phages of *S. flexneri* revealed that SfIV is similar to phage SfII and SfV. The comparative analysis also revealed that SfIV phage contained five proteins which were not found in any other phages of *S. flexneri.* These proteins were: a tail fiber assembly protein, two hypothetical proteins with no clear function, and two other unknown proteins which were encoded by *orfs* present on a moron, that presumably got introduced in SfIV genome from another species via a transposon. These unique proteins of SfIV may play a role in the pathogenesis of the host.

**Conclusions:**

This study reports the isolation and complete genome sequence analysis of bacteriophage SfIV. The SfIV phage has a host range significantly different from the other phages of *Shigella*. Comparative genome analysis identified several proteins unique to SfIV, which may potentially be involved in the survival and pathogenesis of its host. These findings will further our understanding on the evolution of these phages, and will also facilitate studies on development of new phage vectors and therapeutic agents to control infections caused by *S. flexneri.*

## Background

*Shigella* diarrhoeal illness continues to be a leading cause of morbidity and mortality worldwide, particularly in the developing countries. Annually there are estimated 7 million cases of shigellosis globally, of which 1.1 million result in death. *Shigella* encompasses four subgroups (*S. boydii, S. dysenteriae, S. flexneri,* and *S. sonnei*) [1]. Although *S. dysenteriae* type 1 is associated with the most severe form of the disease and high mortality rate when epidemics occur, most of the deaths are attributed to the endemic form of the disease, which is most often caused by *S. flexneri*[2]*.*

Based on the O-antigen structure of the lipopolysaccharide (LPS) molecule, *S. flexneri* is currently divided into 17 serotypes [3-5]. All the serotypes, with an exception of serotype 6, share a common O-antigen backbone comprising of repeating tetrasaccharide units (*N*-acetylglucosamine attached to three rhamnose residues) [6]. Modifications to the basic O-antigen backbone, which occur through glucosylation and/or *O*-acetylation of one or more sugars within the repeating unit, form the basis of serotype conversion. *O*-acetylation is known to occur only on rhamnose III residue, while glucosylation can occur on any of the three rhamnoses or on the *N*-acetylglucosamine. The genes which mediate the addition of glucosyl or *O*-acetyl residues to the specific sugars are encoded by temperate bacteriophage. These phage are integrated into the host chromosome, forming a prophage that stably expresses serotype conversion genes [7].

The glucosylation loci in these phages contain three genes [*gtrA*, *gtrB*, and *gtr*(type)] that are arranged in a single operon known as a *gtr* cluster. The first two genes, *gtrA* and *gtrB,* which encode proteins involved in transferring the glucosyl group from cytoplasm into the periplasm, are highly conserved and interchangeable between different serotypes. Whereas, the third gene, *gtr*(type) is unique to each serotype and encodes a glucosyltransferase that is responsible for attaching the glucosyl group to a specific sugar unit of the O-antigen via a specific linkage [8-14]. In contrast, the addition of an *O*-acetyl group is mediated by only one protein, an *O*-acetyltransferase encoded by the *oac* gene of phage Sf6 [15].

Five serotype converting bacteriophages: SfI, SfII, SfV, SfX and Sf6, have previously been isolated and studied [12,15-19]. Lysogenisation of these bacteriophages have been shown to convert serotype Y strains to serotype 1a, 2a, 5a, X and 3b, respectively, and the *gtrI*, *gtrII*, *gtrV*, *gtrX*, and *oac* genes, which are involved in the serotype conversion have been characterized. Analysis of the phage genomes has also revealed that in all these phages, the genes involved in serotype conversion are located adjacent to the *int attP* region, and the phage DNA (except Sf6) has integrated into the *thrW* gene, in the *pro*–*lac* region of the host genome [7].

The type IV O-antigen modification genes from *S. flexneri* 4a strains have been characterized and shown to convert serotype Y strain to serotype 4a. Also, analysis of the fragment of DNA upstream of the *gtrA* gene in serotype 4a strain has revealed the presence of bacteriophage *int* gene and *attP* site [8]. However, attempts to isolate a serotype converting SfIV bacteriophage have not been successful to date.

In this study, we report the isolation and characterization of bacteriophage SfIV, induced from a wildtype *S. flexneri* 4av strain. The complete sequence of phage SfIV was determined and compared to other known *Shigella* serotype converting phages at the protein level. Our results suggest that the SfIV phage was similar to SfII and SfV phages, and there was a considerable conservation in the genomic architecture of all the phages. Five proteins identified were unique to SfIV, four of which could potentially play a role in pathogenesis of its host.

## Methods

### Bacterial strains, phage and media

Bacteriophage SfIV was isolated from *S. flexneri* 4av strain (SFL1522, International Centre for Diarrhoeal Disease Research, Bangladesh) using the UV irradiation protocol described by Adams et al. [8]. Plaque assays were performed on the lysate after induction. Bacteriophage stocks were prepared by picking a single plaque, propagating on serotype Y strain (SFL124) [20], and precipitating phage using polyethylene glycol, as described in Sambrook et al. [21].

Phage was routinely propagated in NZCYM broth and *S. flexneri* strains were grown in Luria–Bertani (LB) broth or LB agar.

### Electron microscopy

For electron microscopy, phage preparations were negatively stained with 2% phosphotungstic acid (pH 7.0) and electron micrographs were taken with a Hitachi H600 transmission electron microscope.

### Determination of phage host range and lysogens

To determine the host range of bacteriophage SfIV, different dilutions of the purified phage stock were made in SM buffer (100 mM NaCl, 50 mM Tris–HCl pH 7.5, 8 mM MgSO_4_ and 0.002% (w/v) gelatin). 100 μl of overnight culture of the required bacterial host strain was then spread on the LB agar plate. Once the plates were dry, 5–10 μl of the phage stock of different dilutions was spotted on the plate. The plates were incubated overnight at 37°C and were examined for appearance of the clear zones around the phage drop.

Lysogens of SfIV were obtained using the method previously described by Marvis et al. [12].

### Genomic DNA purification and sequencing

Bacterial genomic DNA was isolated using GE Healthcare genomic DNA isolation kit (GE Healthcare), according to the manufacturer’s instructions. Complete genome of the host strain SFL1522 was sequenced using 250 bp paired-end, Miseq, Illumina sequencing (performed at the Ramaciotti Centre, University of New South Wales). Reads generated were then assembled into contigs using CLC Genomics Workbench (Ver 5.5.1, CLC Bio). The contigs containing the *gtrA* and the *int* gene were extracted. The gaps between the desired contigs were filled by designing primers binding to the end of each contig and amplifying the segment using SFL1522 genomic DNA. PCR product obtained was further purified using the gel purification kit (Promega) according to the manufacturer’s instructions. Purified PCR product was then sequenced using the ABI Prism BigDye Terminator Cycle Sequencing Ready reaction kit, and the reactions were performed in a GeneAmp 2400 thermal cycler (Perkin Elmer) according to the manufacturer’s protocol. Reactions were run on an ABI Prism 377 Automated sequencer at the Biomedical Resources Facility, John Curtin School of Medical Research, Australian National University.

### Sequence assembly and analysis

The contigs were assembled using CLC Genomics Workbench (Ver 5.5.1, CLC Bio) and the open reading frames (*orfs*) were determined using the NCBI ORF finder program and CLC Main Workbench (Ver 6.6.2, CLC Bio). The genome was scanned for tRNA using tRNAscan-SE Search server [22], and the Rho-independent terminators were identified using ARLOND terminator finding program [23].

### Genome comparison

Multiple genome alignment was performed using Mauve [24], and the protein level alignments were perfomed using ClustalW [25]. The accession numbers for the phages used for comparative genomics were: Enterobacteria phage SfI (JX509734), *Shigella* phage SfII (NC_021857), Enterobacteria phage SfV (NC_003444), phage SfX (NC_017328), *Shigella* phage Sf6 (NC_005344), Enterobacteria phage cdtI (NC_009514), Enterobacteria phage lambda (NC_001416), Enterobacteria phage phiP27 (NC_003356), Enterobacteria phage Mu (NC_000929), StxI phage (AP005153), *Salmonella* phage epsilon34 (NC_011976), *Salmonella* phage ST64B (NC_004313), *Salmonella* phage ST64T (NC_004348), Enterobacteria phage ST104 (NC_005841), and *Salmonella* phage SE1 (DQ003260).

### Availability of supporting data

The nucleotide sequence of SfIV phage reported in this article has been deposited in the GenBank database as accession number KC814930.

## Results and discussion

### Isolation of bacteriophage SfIV

SfIV phage was induced from serotype 4av, strain SFL1522. Clear plaques were produced when the lysate after induction from SFL1522 was plated onto the indicator strain SFL124 (serotype Y). The colonies isolated from turbid areas of the plaques were assessed by slide agglutination, PCR and SfIV infection. Colonies showing positive agglutination with both: group 3,4 (specific for serotype Y) and type IV antisera (specific for serotype IV), were assumed to be lysogens. Results of PCR amplification and SfIV infection indicated that the lysogens contained the SfIV-specific *gtrIV* gene and were resistant to SfIV infection.

### Morphology of SfIV

**Figure 1:**
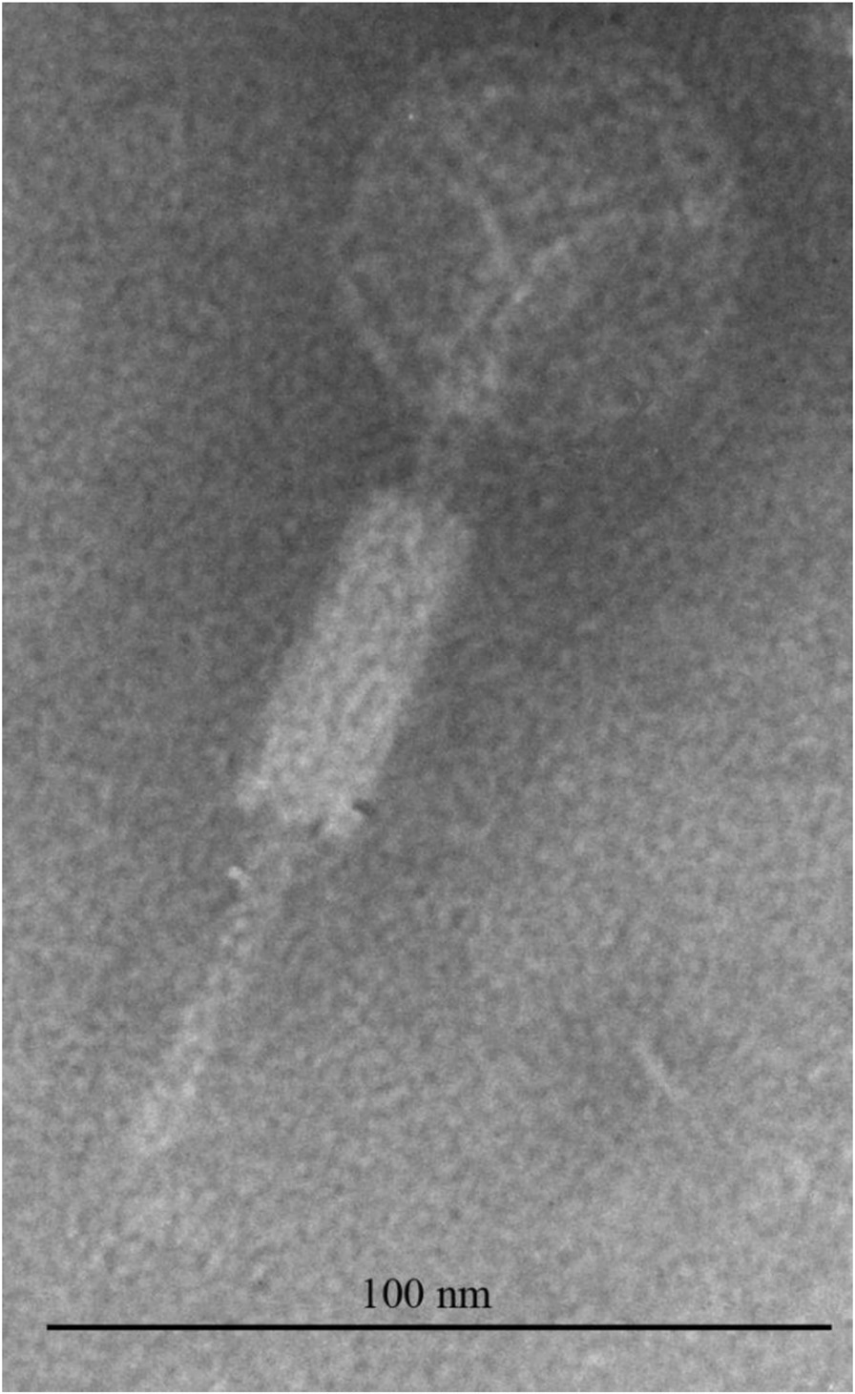
**Electron micrograph of bacteriophage SfIV.** Electron micrograph of phage SfIV, stained with 2% phosphotungstic acid. Bar 100 nm.

Electron microscopy was used to determine bacteriophage SfIV morphology. Electron micrographs revealed that the SfIV phage particle has an isometric head of ca. 55 nm and a long contractile tail of ca. 101 nm (Figure 1). It is a typical group 'A’ phage, of the family *Myoviridae*, and order *Caudovirales*, according to the morphological classification of Bradley [26]. Appearance of SfIV also resembles other serotype converting phages of *Shigella,* like SfV [17], SfII [12] and a newly isolated phage SfI [19].

### Host range of phage SfIV

The host range of the SfIV bacteriophage was determined with twelve serotypes (1a, 1b, 1c, 2a, 2b, 3a, 3b, 4a, 4b, 5a, X, and Y) of *S. flexneri*. Of the serotypes examined, SfIV was capable of infecting serotypes: 1a, 1b, 1c, X and Y. This suggests that, in comparison to other morphologically similar *Shigella* phages, SfIV has a much broader host range than SfI (infects only X and Y serotypes) [19], and SfII (infects serotypes 3a, 5a and Y), but not SfV which infects seven serotypes (1a, 1b, 2a, 2b, 3b, 4b, and Y) [27]. Surprisingly, there is very little similarity between the host range of these bacteriophages, with serotype Y being the only common serotype infected by all the four phages.

### Determination of the phage SfIV genomic sequence

The complete DNA sequence of bacteriophage SfIV was determined using a combination of Illumina (Miseq) and PCR sequencing. High throughput sequencing of the host (SFL1522) DNA, generated 4,32,905 pairs of reads, which is about 25 fold genome coverage. The genomic sequence was then de-novo assembled into 700 contigs and scaffolds using the CLC Genomics Workbench (Ver 5.5.1, CLC Bio). The accuracy of the CLC assembly was further verified using another assembly tool, Velvet [28], and by mapping the reads back to the contigs.

SfIV phage genome was distributed between two contigs of 27 kb and 14 kb. The gap between the two contigs was then closed by targeted PCR and subsequent sequencing, yielding a contiguous prophage SfIV genome flanked by the *int* and *gtrA* gene. 46 bp *attp* site (identical to phage SfI and SfV) was identified on either ends of the prophage DNA, and was used to construct a circular SfIV bacteriophage genome. The linear orientation of the sequence starting from small terminase gene and ending with the *cos* site was then used for further analysis.

**Figure 2:**
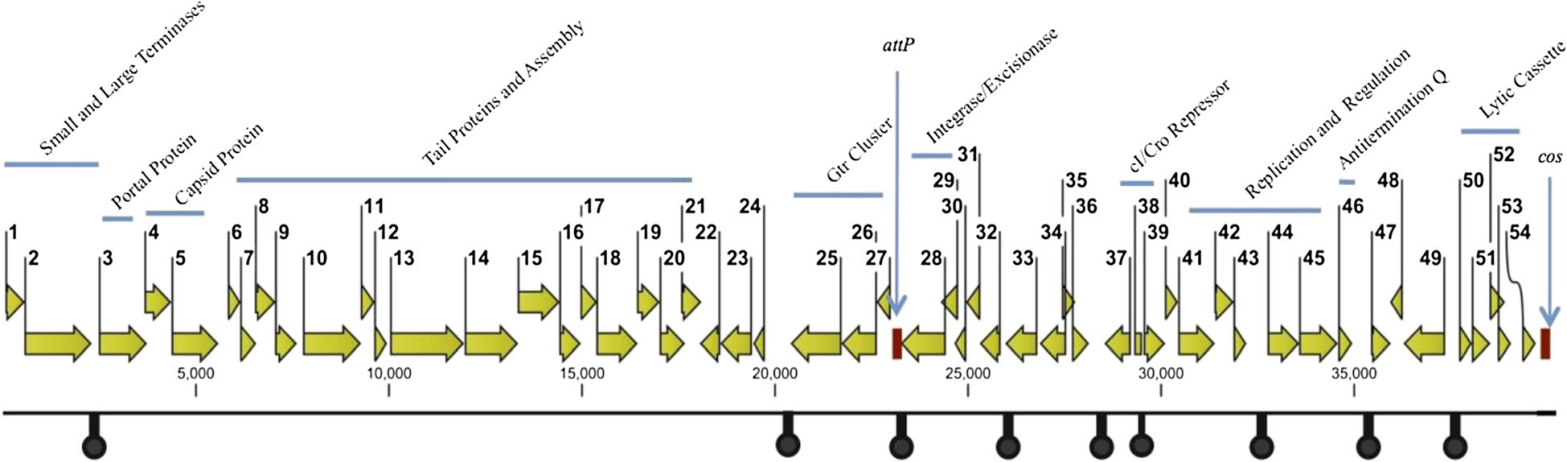
**Genetic map of bacteriophage SfIV.** The SfIV phage genome is shown with a scale in bp. The arrows above the scale represent predicted open reading frames, with the arrowheads indicating the direction of transcription. Putative function and names of genes are given above. The *attP/cos* site is indicated in red. The Rho-independent terminators are indicated as black knobs below the scale.

The genome of SfIV consists of 39,758 bp with an average GC content of 50.29%, which is very similar to its host (50.90%). The bioinformatics analysis revealed a total of 54 *orfs*, taking up to 90.05% of the coding capacity of this virus. A plausible Shine-Dalgarno sequence and a start codon (44 with ATG, 9 with GTG and 1 with TTG) were identified for all the *orfs*. Thirty eight putative SfIV genes are transcribed rightwards (on the genetic map), while sixteen genes transcribed leftwards (Figure 2). The protein corresponding to each *orf* was then compared with the non-redundant protein databases using the NCBI BlastP tool. The database search yielded matches with other proteins with identity values between 33% to 100% (Additional file 1: Table S1). The genome of bacteriophage SfIV was also scanned for the presence of tRNA’s and Rho-independent termiantors. The predicted Rho-independent terminators are summarized in Figure 2, however, no tRNA genes were identified.

### Comparison of SfIV with other serotype converting phages of *S. flexneri*

**Figure 3:**
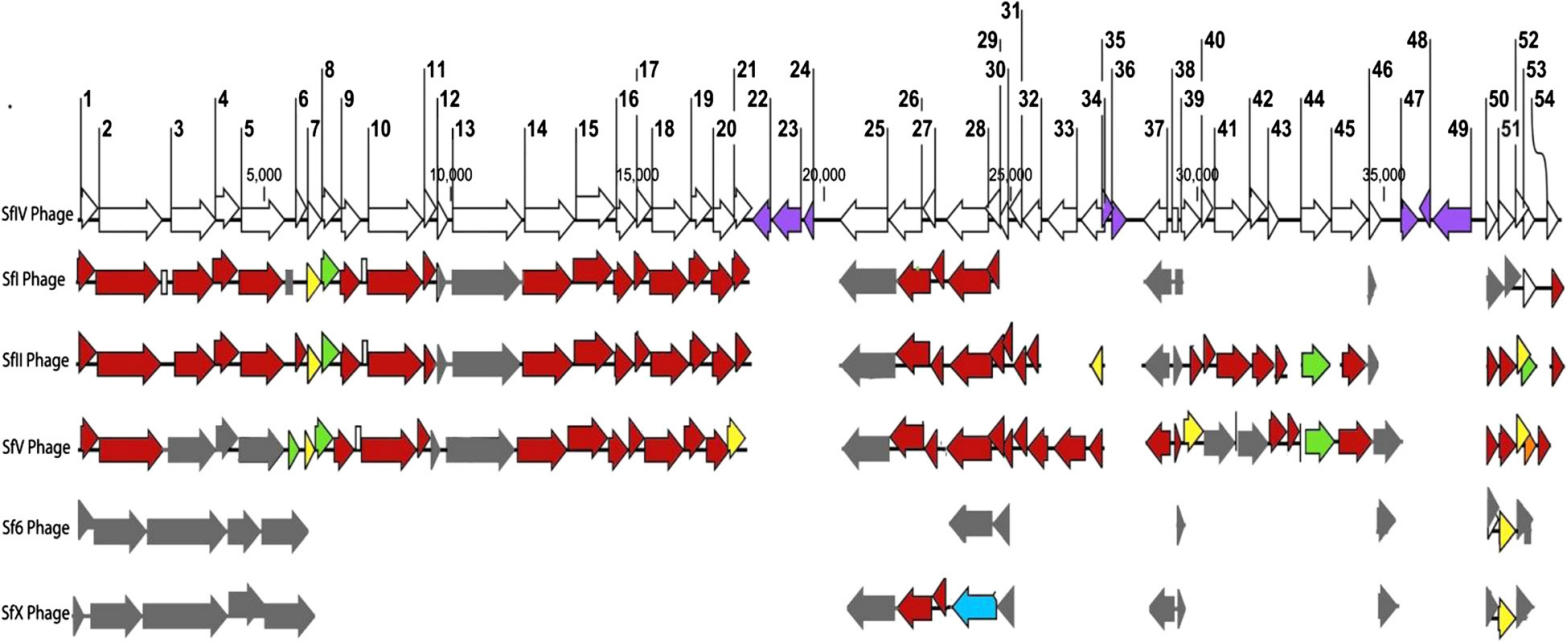
**Comparison of SfIV with other serotype converting phages of *S. flexneri.*** Protein corresponding to each *orf* was compared with its equivalent protein in SfI, SfII, SfV, Sf6 and SfX phages using ClustalW. The arrows below the SfIV map are colour coded and represent the amino acid sequence identity between the SfIV proteins and their equivalents in other phages. The colour codes are:- red: 90-100% identity, yellow: 80-89% identity, orange: 70-79% identity, green: 60-69% identity, blue: 50-59% identity and grey: 10-50% identity. The purple arrows in the SfIV map represent the unique proteins of phage SfIV.

The genome of bacteriophage SfIV was first compared to 15 previously sequenced phages from various hosts: *S. flexneri* (5), *E. coli* (5), and *Salmonella* (5), using Mauve (Additional file 2: Figure S1). The results of multiple alignment revealed that the SfIV bacteriophage had high degree of DNA sequence similarity with other serotype converting bacteriophages of *S. flexneri* in particular SfI, SfII and SfV, but not with SfX and Sf6. Considerable similarity was also obtained with two *E. coli* phages: phage phiP27 and phage cdtI, and one *Salmonella* phage ST64B. Rest of the lambdoid phages showed limited silmilarity to SfIV, with phage Mu showing no similarity at all. To get a better understanding of the degree of relatedness among the *Shigella* phages, bacteriophage SfIV encoded proteins were compared with those of other serotype converting phages of *S. flexneri* (Figure 3).

*orf-1* and *orf-2*, encode the phage SfIV small and large terminase subunits, respectively, which are required for packaging bacteriophages dsDNA into the cavity of preformed protein shells called proheads [29]. The terminases of bacteriophage SfIV show high amino acid level identity (>90%, Figure 3) with the terminases of bacteriophage SfI, SfII and SfV, suggesting that the packaging mechanism is same in these phages. This was verified by the genome sequence analysis of phage SfIV which revealed a *cos* site identical to phage SfI, SfII and SfV. Thus, like SfI, SfII, and SfV, phage SfIV uses bacteriophage lamba like packaging in which virion genomes are generated by *cos* site specific DNA cleavage at the beginning and at the termination of every packaging event. This results in all the virions with DNA of identical, specific ends, generating only precise fragments on restriction digestion. Furthermore, very limited similarity observed between the terminases of SfIV, Sf6 and SfX could be because of the different packaging mechanisms used by Sf6 and SfX. The packaging mechanism of bacteriophage Sf6 and SfX is like phage P22, in which the terminases bind to the *pac* recognition site, and the DNA is packaged by the headful mechanism [15,29,30].

Databases homology searches suggested that *orf-3*, *orf-4* and *orf-5* encode the bacteriophage portal protein, prohead protease and major head protein, respectively (Additional file 1: Table S1). Protein alignment results have shown that these three proteins of SfIV share >90% identity with their counterparts in phage SfI and SfII, but very little identity was obtained with those of SfV. However, it should be noted that even though the SfV proteins show little amino acid similarity, they still belong to the HK97 family of proteins (like SfI, SfII and SfIV). Thus, like several other proteins from tailed bacteriophages, these could be divergent homologues showing conserved structure and function [31]. In contrary, viral capsid assembly proteins in bacteriophage Sf6 and SfX are different. The coat protein of Sf6 is similar to that of bacteriophage P22 and is known to include surface exposed domain (a conserved feature specific to P22 like bacteriophages) with an “HK97-like” core [32]. In addition, Sf6 bacteriophage also contains a separate scaffold protein and three other head completion genes [15].

Analysis of proteins encoded by *orf-6* through *orf-21* suggests that this region of the SfIV genome is involved in bacteriophage tail structure and assembly (Additional file 1: Table S1). The major difference between the tail proteins of SfIV and other 3 phages (SfI, SfII and SfV) was ORF-12, ORF-13 and ORF-22. While ORF-12 (a hypothetical protein) and ORF-13 (a tail tape measure protein) of SfIV showed <50% identity with SfI (ORFs 15 and 16), SfII (ORFs 15,16), and SfV (ORFs 13,14), ORF-22 which is a predicted tail fiber assembly protein was completely missing in SfI, SfII and SfV. Since phage tail fibers bind to the receptors on the bacterial host for adsorption [33], presence of an additional tail fiber assembly protein in SfIV could explain the fact that the host range of SfIV does not match with the above mentioned three phages. Unlike SfI, SfII, SfIV, and SfV (which belong to group A1), phage Sf6 and SfX are members of class C bacteriophages, consisting of icosahedral head and a short tail. Thus, unlike SfIV, Sf6 and SfX have only one protein in the tail spike module [34].

SfIV phage *orfs 25–27* encode for the O-antigen modification cluster. o*rf-25* corresponds to the *gtrIV* gene, the serotype specific modification gene [8]. *orfs* 26 and 27, encode for the *gtrB*_*IV*_ and *gtrA*_*IV*_, respectively. As expected, no protein level similarity was observed between the type specific modification genes of the six bacteriophages. However, the *gtrA* and *gtrB* genes were highly conserved (with >90% protein level identity). The integrase and excisionase proteins encoded by *orf-28* and *orf-29* of SfIV were also conserved between SfI, SfII, SfIV and SfV. However, when compared with the Int and Xis of Sf6 and SfX, very little identity was observed with proteins of both Sf6 and SfX phage. Different Int protein of Sf6 could explain its integration into *agr*W, instead of *thr*W tRNA gene [7].

*orfs 30–34* encode for hypothetical proteins with no clear functions. While these proteins were completely missing in SfI, Sf6 and SfX, the same cluster of unknown genes was observed in SfV *(orfs 28–32)* and was found to share >98% protein level identity with SfIV proteins. The homologues of ORFs -30, -31 and -32 were present in SfII as well (ORFs 32, 33 and 34), however, ORF-33 of SfIV was missing in SfII, and ORF-34 showed only 85% identity with ORF35 of SfII.

In all the lambdoid phages, the regulatory switch that determines whether the phage will follow a lytic or lysogenic cycle includes an operator/promoter sequence and two major proteins similar to the Cro and cI repressor proteins of phage lambda. Analysis of the SfIV genome revealed that the *orfs -37* and *-38* encode for the cI and Cro proteins, which are 100% identical with the cI and Cro of SfV. The cI and Cro proteins of SfI, SfII, Sf6 and SfX, however, showed very limited identity to SfIV. Analysis of the SfIV sequence for additional lambda regulatory elements CII, cIII, or N proteins, revealed that like SfII and SfV, SfIV does not contain any additional regulatory factors, although these factors were present in the other three phages (SfI, Sf6 and SfX).

The blastp search results have also revealed that the SfIV *orf -39* and -*40* encode for a putative DNA-binding regulatory protein, and an unknown protein, which had >90% identity to SfII ORFs 39 and 40 proteins. The replication module of SfIV which stretches from *orf-41* to *orf-45*, showed high identity to the corresponding proteins of SfII and SfV, but was not present in the SfI, Sf6 and SfX genome sequences. Intrestingly, most of the replication module of SfIV was similar to SfII and SfV, however, the methylase gene present in SfII and SfV replication module was not found in SfIV.

The antitermination Q protein encoded by *orf-46* regulates the expression of lamdoid phage lytic proteins by modifying the RNA polymerase near the phage late gene promoter allowing it to pass one or more terminators [35]. The antitermination Q protein of SfIV was very different from all the other phages, sharing <50% identity with its equivalents. The lytic cassette of SfIV consists of holin, lysin, Rz and Rz1 proteins (encoded by *orfs 50–53*). These proteins share high degree of identity with their counterparts in SfII and SfV, but are different to their equivalents in SfI, Sf6 and SfX. Like bacteriophage lambda, the lytic cassette of Sf6 and SfX contains holin with a dual start motif (resulting in production of holin and anti-holin by the same gene) that regulates the timing of lysis [36]. However, the mechanism of controlling the timing of lysis in SfI, SfII, SfIV and SfV is not known. *orf-54* the last gene of SfIV encodes for a HNH endonuclease domain protein which had homologues in SfI, SfII, and SfV, but not in Sf6 and SfX. Given that the *orf-54* is a member of phage late genes and its homologues were only observed in the *Shigella* phages lacking the dual start motif of holin, it could be hypothesized that this protein plays a role in controlling the timing of lysis.

### Proteins unique to phage SfIV

There were several proteins in the SfIV genome (highlighted in purple, Figure 3), which did not have homologues in any other serotype converting phages of *S. flexneri*. These are: ORF-22, a tail fiber protein (discussed earlier), ORFs -23, -24 identified as insertion elements ISEhe3 Orf B, ISEhe3 Orf A, respectively, and ORFs -35 and -36 proteins with no clear function. These two unknown proteins are potential candidates for future virulence studies as several bacteriophages are known to encode factors that alter host properties relevant to various stages of infection [37-39]. The region between antitermination Q and lytic cassette (o*rfs 47–49*) was identified as a moron. Like a typical moron, this region is flanked by transcription initiation and termination signals. Moreover, analysis of the GC content has revealed that this region has a significantly lower GC content (39.20%) than the rest of the SfIV phage (50.29%). *orf-47* encodes for a transposase (IS1 Transposase B). Based on this, we hypothesize that *orf-48* and *orf-49*, came with *orf-47* in the SfIV genome from another species. As morons are expressed in lysogens and are known to confer selective advantage to the host [16], it is reasonable to assume that the unknown proteins encoded by *orfs -48* and -*49* could be involved in the pathogenesis of its host bacterium.

## Conclusions

This is the first report on isolation of a serotype converting bacteriophage SfIV from a wildtype *S. flexneri* 4av strain. The complete genome sequence of phage SfIV was determined, and the comparison between SfIV and other *S. flexneri* serotype converting phages was obtained at the protein level. The analysis revealed that the SfIV phage was more closely related to SfII and SfV, than the rest of the phages. Nevertheless, SfIV phage was found to contain five proteins which were not present in any other phages of *S. flexneri* and may potentially be involved in the pathogenesis of its host. Moreover, the comparison study also indicated that the general organization of the genes was similar in all the phages. Further molecular biological studies based on the comparative genomic analyses of these phages will be useful in elucidating the evolution of these related phages. It will also provide essential information for the development of new phage vectors and therapeutic agents to control infection caused by *S. flexneri*.

## Competing interest

The authors declare that they have no competing interests.

## Authors’ contributions

RJ contributed to the experimental design of the study, carried out all experiments, analysis and drafted the manuscript. KAT provided the *S. flexneri* 4av strain. NKV conceived and directed the study, participated in its experimental design, analysis of the results and critically revised the manuscript. All authors read and approved the final manuscript.

## Supplementary Material

Additional file 1: Table S1 Analysis of predicted *orfs* and proteins of SfIV. Click here for file

Additional file 2: Figure S1 DNA sequence alignment of SfIV phage with other related phages. The alignment of SfIV phage with other phages from *S. flexneri, E. coli* and *Salmonella*. The results obtained using Progressive Mauve with default parameters showing high degree of similarity of SfIV with *S. flexneri* phage SfII, SfV and SfI. Coloured outlined blocks surround regions of the genome that aligned to part of another genome. The degree of DNA sequence similarity is indicated by the height of the coloured bars inside the blocks. Click here for file
