# Supplementary material for: Isolation, characterization and comparative genomics of bacteriophage SfIV: a novel serotype converting phage from Shigella flexneri
Source: BMC Genomics. 2013 Oct 3;14:677. doi: 10.1186/1471-2164-14-677 (PMC3851460; doi:10.1186/1471-2164-14-677)
Supplement: Additional file 1: Table S1 — Analysis of predicted orfs and proteins of SfIV. [file 1471-2164-14-677-S1.docx]

**Table 1: Analysis of predicted *orfs* and proteins of SfIV.**

| **Gene name (*orf*)** | **Funtion** | **Region** | **Gene product size** | **Related phage and bacterial proteins** | **GenBank accession no.** | **BlastP e values (% positives)** |
| --- | --- | --- | --- | --- | --- | --- |
| 1 | Small terminase subunit | 68..562 | 164 | phage terminase, small subunit, P27 family [164aa, Escherichia coli 2.4168]; putative phage terminase, small subunit, P27 family [164aa, Escherichia coli E22] | [ZP_12061435.1, ZP_03045385.1](http://www.ncbi.nlm.nih.gov/protein/417274091?report=genbank&log$=prottop&blast_rank=1&RID=FEE7ZFK3014) | 6E-115 (100), 2e-114 (100) |
| 2 | Large terminase subunit | 559..2292 | 577 | bacteriophage V large terminase subunit [577aa, Shigella sp. D9]; phage terminase [Escherichia coli KTE212] | [ZP_08393216.1](http://www.ncbi.nlm.nih.gov/protein/332280803?report=genbank&log$=prottop&blast_rank=1&RID=FEEPZSYT015) | 0.0 (100) |
| 3 | Phage portal protein | 2486..3727 | 413 | HK97 family phage portal protein [413aa, Escherichia coli KTE25]; HK97 family phage portal protein [413aa, Escherichia coli KTE212] | [ZP_19638943.1](http://www.ncbi.nlm.nih.gov/protein/432396151?report=genbank&log$=prottop&blast_rank=1&RID=FEVKN6ED015) | 0.0 (100) |
| 4 | Prohead protease | 3669..4355 | 228 | pro-head protease [228aa, Escherichia coli APEC O1]; pro-head protease [228aa, Shigella sp. D9] | [YP_541969.1](http://www.ncbi.nlm.nih.gov/protein/91211983?report=genbank&log$=prottop&blast_rank=1&RID=FEXCFM7101R) | 1E-163 (100) |
| 5 | Major head protein | 4370..5575 | 401 | phage major capsid protein, HK97 family [401aa, Shigella flexneri 6603-63]; phage capsid family protein [401aa, Escherichia coli H120] | [ZP_12878547.1](http://www.ncbi.nlm.nih.gov/protein/418253522?report=genbank&log$=prottop&blast_rank=1&RID=FF97V1NX01R) | 0.0 (100) |
| 6 | Unknown | 5827..6150 | 107 | hypothetical protein SF660363_0336 [107aa, Shigella flexneri 6603-63]; bacteriophage protein [107aa, Shigella flexneri 5 str. 8401] | [YP_687822.1](http://www.ncbi.nlm.nih.gov/protein/110804302?report=genbank&log$=prottop&blast_rank=1&RID=FFAGH87701R) | 1E-72 (100) |
| 7 | Unknown | 6147..6557 | 136 | putative phage head-tail adaptor [136aa, Shigella flexneri 6603-63]; putative phage head-tail adaptor [136aa, Escherichia coli DEC12A] | [ZP_12878550.1, YP_006117877.1](http://www.ncbi.nlm.nih.gov/protein/418253525?report=genbank&log$=prottop&blast_rank=1&RID=FFAYJSV601R) | 2E-95 (100), 3e-92 (97) |
| 8 | Unknown | 6529..7053 | 174 | hypothetical protein WE9_05112 [174aa, Escherichia coli KTE21]; hypothetical protein ECDEC12B_5453 [174aa, Escherichia coli DEC12B] | [ZP_19637586.1, ZP_13867555.1](http://www.ncbi.nlm.nih.gov/protein/432394776?report=genbank&log$=prottop&blast_rank=1&RID=FH98BJ7G01R) | [4e-122, 3e-120 (100)](http://www.ncbi.nlm.nih.gov/protein/432394776?report=genbank&log$=prottop&blast_rank=1&RID=FH98BJ7G01R) |
| 9 | Unknown | 7050..7610 | 186 | hypothetical protein SF660363_0339 [186aa, Shigella flexneri 6603-63]; hypothetical protein EC50588_2434 [186aa, Escherichia coli 5.0588] | [ZP_12878552.1, ZP_11978781.1](http://www.ncbi.nlm.nih.gov/protein/418253527?report=genbank&log$=prottop&blast_rank=1&RID=FHB5Z6TD01R) | 2e-132 (99) , 3e-132 (99) |
| 10 | Tail sheath protein | 7773..9269 | 498 | bacteriophage Mu tail sheath family protein [498aa, Shigella flexneri 6603-63]; phage tail sheath protein [498aa, Escherichia coli KTE25] | [ZP_12878554.1, ZP_19638952.1](http://www.ncbi.nlm.nih.gov/protein/418253529?report=genbank&log$=prottop&blast_rank=1&RID=FHBS0UHH01R) | 0.0 (99), 0.0(99) |
| 11 | Unknown | 9269..9625 | 118 | hypothetical protein SfVp12 [118aa, Enterobacteria phage SfV]; phage tail protein [118aa, Escherichia coli S88] | [NP_599044.1](http://www.ncbi.nlm.nih.gov/protein/19549002?report=genbank&log$=prottop&blast_rank=1&RID=FHR6XYVF01R) | 1e-82 (100) |
| 12 | Unknown | 9622..9945 | 107 | hypothetical protein SF660363_0343 [107aa, Shigella flexneri 6603-63], bacteriophage protein [107aa, Shigella sp. D9], sigma factor domain protein [107aa, Escherichia coli TW07793] | ZP_12878556.1, YP_002411656.1, ZP_12074976.1 | 3E-70 (100), 7e-70 (100), 7e-69 (99) |
| 13 | Phage tail tape measure protein | 10030..11937 | 635 | phage tail tape measure protein, TP901 family, core region [635aa, Escherichia coli DEC7B]; phage tail tape measure protein, TP901 family, core region [635aa, Shigella flexneri 6603-63] | [ZP_13717508.1, ZP_12878557.1](http://www.ncbi.nlm.nih.gov/protein/419173650?report=genbank&log$=prottop&blast_rank=1&RID=FHSFMGNF015) | 0.0 (99), 0.0 (99) |
| 14 | Tail/DNA circulation protein | 11962..13338 | 458 | phage tail/DNA circulation protein [458aa, Escherichia coli KTE18]; phage tail/DNA circulation protein [458aa, Escherichia coli KTE23] | [ZP_19961285.1](http://www.ncbi.nlm.nih.gov/protein/432726402?report=genbank&log$=prottop&blast_rank=1&RID=FHSYMZPZ015) | 0.0 (99) |
| 15 | Tail protein | 13335..14414 | 359 | bacteriophage Mu P protein [359aa, Shigella flexneri 2850-71]; phage tail protein [359aa, Escherichia coli KTE25] | [ZP_07105019.1](http://www.ncbi.nlm.nih.gov/protein/300824918?report=genbank&log$=prottop&blast_rank=1&RID=FHU0JSDZ015) | 0.0 (100) |
| 16 | Baseplate assembly V family protein | 14414..14962 | 182 | phage baseplate assembly V family protein [182aa, Shigella flexneri 6603-63], phage baseplate assembly protein V [182aa, Escherichia coli KTE25] | [ZP_12878560.1](http://www.ncbi.nlm.nih.gov/protein/418253535?report=genbank&log$=prottop&blast_rank=1&RID=FHUPPXFE015) | 2E-130 (100) |
| 17 | Tail protein | 14962..15387 | 141 | tail protein [142aa, Escherichia fergusonii ATCC 35469]; tail protein [141aa, Enterobacteria phage SfV] | [YP_002381806.1; NP_599050.1](http://www.ncbi.nlm.nih.gov/protein/218548015?report=genbank&log$=prottop&blast_rank=1&RID=FHVBFCXT01R) | 1E-96 (100), 2e-96 (100) |
| 18 | Baseplate protein | 15374..16432 | 352 | baseplate J-like family protein [352aa, Shigella flexneri 6603-63]; phage baseplate protein [352aa, Escherichia coli KTE54] | [ZP_12878562.1, ZP_20008241.1](http://www.ncbi.nlm.nih.gov/protein/418253537?report=genbank&log$=prottop&blast_rank=1&RID=FHW9479N015) | 0.0 (100), 0.0 (99) |
| 19 | Tail protein | 16423..17007 | 194 | tail protein [194aa, Shigella flexneri 6603-63], phage tail protein [194aa, Escherichia coli KTE18] | [ZP_12878563.1, ZP_19961290.1](http://www.ncbi.nlm.nih.gov/protein/418253538?report=genbank&log$=prottop&blast_rank=1&RID=FHWJ8FX701R) | 2E-141 (100), 3e-140 (99) |
| 20 | Unknown | 17011..17661 | 216 | hypothetical protein SF660363_0351 [216aa, Shigella flexneri 6603-63], hypothetical protein WE3_00836 [216aa, Escherichia coli KTE18] | [ZP_12878564.1, ZP_19961291.1](http://www.ncbi.nlm.nih.gov/protein/418253539?report=genbank&log$=prottop&blast_rank=1&RID=FHWYJ6DX015) | 2E-156 (100), 2e-154 (98) |
| 21 | Tail fiber assembly protein | 17570..18085 | 171 | putative tail fiber assembly protein (P37) [171aa, Escherichia coli M605], phage tail fiber protein [165aa, Shigella flexneri] | [ZP_08347206.1, AFA52229.1](http://www.ncbi.nlm.nih.gov/protein/331646103?report=genbank&log$=prottop&blast_rank=1&RID=FHY94EF401R) | 7E-118 (97), 3e-108 (94) |
| 22 | Tail fiber assembly protein | complement(18057..18581) | 174 | caudovirales tail fiber assembly family protein [174aa, Shigella flexneri 6603-63], phage tail fiber assembly protein [200aa, Escherichia coli KTE180] | [ZP_12878566.1, ZP_19809846.1](http://www.ncbi.nlm.nih.gov/protein/418253541?report=genbank&log$=prottop&blast_rank=1&RID=J6BY8ZKZ01R) | 2E-122 (100), 2e-113 (97) |
| 23 | Transposase B/ISEhe3 (orfB) | complement(18589..19401) | 270 | integrase core domain protein [270aa, Shigella flexneri 6603-63], Transposase subunit B [270aa, Salmonella enterica subsp. enterica serovar Senftenberg str. SS209] | ZP_12878567.1 , ZP_16317879.1 | 0.0 (100), 0.0 (96) |
| 24 | Transposase A/ISEhe3 (orfA) | complement(19439..19735) | 98 | transposase family protein [98aa, Shigella flexneri 6603-63], ISEhe3 OrfA [98aa, Escherichia coli H591] | ZP_12878568.1, ZP_08379675.1 | 1e-65 (100), 2e-57 (92) |
| 25 | Serotype specific glucosyltransferase | complement(20408..21721) | 437 | GtrIV-Sf [437aa, Shigella flexneri] | [AAK50574.1](http://www.ncbi.nlm.nih.gov/protein/13957539?report=genbank&log$=prottop&blast_rank=2&RID=KWUGG33601R) | 0.0 (99) |
| 26 | Bactoprenol glucosyltransferase | complement(21723..22637) | 304 | glycosyl transferase, family 2 protein [304aa, Shigella flexneri 6603-63], GtrB-IV [304aa, Shigella flexneri] | \|  \| [ZP_12878570.1](http://www.ncbi.nlm.nih.gov/protein/418253545?report=genbank&log$=prottop&blast_rank=1&RID=KWUH7T57014) \| \| --- \| --- \| | 0.0 (100) |
| 27 | Putative flippase | complement(22634..22996) | 120 | putative flippase [120aa, Enterobacteria phage SfV], flippase [120aa, Enterobacteria phage SfI] | [NP_599057.1](http://www.ncbi.nlm.nih.gov/protein/19549013?report=genbank&log$=prottop&blast_rank=1&RID=KWUSW8R8016) | 4e-79 (100) |
| 28 | Integrase | complement(23258..24421) | 387 | Phage integrase [387aa, Escherichia coli O157:H7 str. EC1212], integrase [387aa, Escherichia coli EC1738] | [ZP_02814724.1](http://www.ncbi.nlm.nih.gov/protein/168789717?report=genbank&log$=prottop&blast_rank=1&RID=FTW4M6S5013) | 0.0 (100) |
| 29 | Excisionase | complement(24298..24741) | 147 | excisionase [147aa, Enterobacteria phage SfV], Excisionase from phage origin [138aa, Escherichia coli CUMT8] | [NP_599059.1, ZP_14465385.1](http://www.ncbi.nlm.nih.gov/protein/19548990?report=genbank&log$=prottop&blast_rank=1&RID=J66FJAVX013) | 6E-104 (99), 2e-97(99) |
| 30 | Unknown | complement(24648..24953) | 101 | hypothetical protein SfVp28 [101aa, Enterobacteria phage SfV], hypothetical protein SF0298 [101aa, Shigella flexneri 2a str. 301] | [NP_599060.1](http://www.ncbi.nlm.nih.gov/protein/19549015?report=genbank&log$=prottop&blast_rank=1&RID=FTJ8S30Z01R) | 8e-67 (100) |
| 31 | Unknown | complement(24953..25315) | 120 | hypothetical protein SfVp29 [120aa, Enterobacteria phage SfV], conserved hypothetical protein; CPS-53 (KpLE1) prophage [120aa, Escherichia fergusonii ATCC 35469] | [NP_599061.1](http://www.ncbi.nlm.nih.gov/protein/19549016?report=genbank&log$=prottop&blast_rank=1&RID=FTJ33G3001R) | 4e-83 (100) |
| 32 | Unknown | complement(25306..25842) | 178 | conserved hypothetical protein [187aa, Escherichia coli MS 107-1], putative uncharacterized protein yfdr [178aa, Escherichia coli chi7122] | ZP_07097453.1, YP_002381761.1 | 3E-130 (100), 5e-130 (100) |
| 33 | Unknown | complement(25970..26794) | 274 | hypothetical protein SF660363_0306 [274aa, Shigella flexneri 6603-63], phage protein [274aa, Escherichia coli KTE51] | [NP_755083.1](http://www.ncbi.nlm.nih.gov/protein/26249043?report=genbank&log$=prottop&blast_rank=1&RID=FTGTZWZ201R) | 0.0 (100) |
| 34 | Unknown | complement(26860..27540) | 226 | hypothetical protein ETEC_0272 [226aa, Escherichia coli ETEC H10407], hypothetical protein EFER_0577 [226aa, Escherichia fergusonii ATCC 35469] | [YP_006113858.1, YP_002381763.1](http://www.ncbi.nlm.nih.gov/protein/387610742?report=genbank&log$=prottop&blast_rank=1&RID=FTG59SRB016) | 2e-164 (99), 5e-164 (99) |
| 35 | Unknown | 27428..27772 | 114 | conserved domain protein [114aa, Escherichia coli MS 119-7], conserved hypothetical protein [114aa, Shigella sp. D9] | [ZP_07103592.1, ZP_08392084.1](http://www.ncbi.nlm.nih.gov/protein/300823462?report=genbank&log$=prottop&blast_rank=1&RID=J67NUSZU01R) | 1E-74(99), 7e-74(98) |
| 36 | Unknown | 27691..28125 | 144 | hypothetical protein EFER_0579 [144aa, Escherichia fergusonii ATCC 35469], predicted protein [144aa, Shigella sp. D9] | [YP_002381765.1](http://www.ncbi.nlm.nih.gov/protein/218547974?report=genbank&log$=prottop&blast_rank=1&RID=FRA8XZXA013) | 7E-102 (100) |
| 37 | Repressor / cI | complement(28538..29212) | 224 | e14 prophage; repressor protein phage e14 [224aa, Escherichia coli DH1], phage repressor [224aa, Escherichia coli UTI89] | [NP_415663.1](http://www.ncbi.nlm.nih.gov/protein/16129108?report=genbank&log$=prottop&blast_rank=1&RID=FR41C4XA01R) | 3E-166 (100) |
| 38 | Cro | 29303..29503 | 66 | repressor [73aa, Escherichia coli EPECa14], repressor [66aa, Enterobacteria phage SfV] | [ZP_11493490.1, NP_599067.1](http://www.ncbi.nlm.nih.gov/protein/415786322?report=genbank&log$=prottop&blast_rank=1&RID=FR985MBS01R) | 1E-40 (100), 1E-40 (100) |
| 39 | DNA binding transcriptional regulator | 29547..30098 | 183 | e14 prophage; predicted DNA-binding transcriptional regulator [187aa, Escherichia coli E128010], hypothetical protein SF660363_0313 [183aa, Shigella flexneri 6603-63] | [ZP_11499213.1, ZP_12061386.1](http://www.ncbi.nlm.nih.gov/protein/415800148?report=genbank&log$=prottop&blast_rank=1&RID=J2Y4VRAC015) | 4E-132 (100), 2e-131 (99) |
| 40 | Unknown | 30095..30433 | 112 | e14 prophage protein [112aa, Escherichia coli O55:H7 str. CB9615], phage protein [112aa, Escherichia coli KTE44] | [YP_003502582.1](http://www.ncbi.nlm.nih.gov/protein/291285764?report=genbank&log$=prottop&blast_rank=1&RID=FR3HE7WR01R) | 9E-77 (99) |
| 41 | Replication protein | 30443..31384 | 313 | phage O protein family [Shigella flexneri VA-6], phage replication protein O [Escherichia coli KTE80], hypothetical protein SF660363_0314 [Shigella flexneri 6603-63] | [YP_002117133.1](http://www.ncbi.nlm.nih.gov/protein/194738373?report=genbank&log$=prottop&blast_rank=1&RID=FR38KJHJ013) | 0.0 (100) |
| 42 | Unknown | 31381..31875 | 164 | hypothetical protein T22_18623 [164aa, Escherichia coli O157:H43 str. T22], phage protein [164aa, Escherichia coli KTE210] | [ZP_13498028.1, ZP_19721573.1](http://www.ncbi.nlm.nih.gov/protein/418945100?report=genbank&log$=prottop&blast_rank=1&RID=FR2UMGPR01R) | 1E-115 (100), 4e-116 (99) |
| 43 | Regulation | 31875..32201 | 108 | lexA DNA binding domain protein [108aa, Shigella flexneri 6603-63], LexA repressor [108aa, Escherichia coli KTE28] | [YP_003502586.1](http://www.ncbi.nlm.nih.gov/protein/291285768?report=genbank&log$=prottop&blast_rank=1&RID=JY55WY9901R) | 5E-73 (100) |
| 44 | KilA-N domain family protein | 32756..33571 | 271 | kilA-N domain protein [271aa, Shigella flexneri 6603-63], KilA-N domain family protein [271aa, Escherichia coli O104:H4 str. 2011C-3493] | [ZP_12878532.1, YP_006778771.1](http://www.ncbi.nlm.nih.gov/protein/418253507?report=genbank&log$=prottop&blast_rank=1&RID=FR1EJV7Y013) | 0.0 (100), 0.0 (99) |
| 45 | Unknown | 33573..34568 | 331 | hypothetical protein EFER_0593 [331aa, Escherichia fergusonii ATCC 35469], hypothetical protein ECIAI39_4867 [331aa, Escherichia coli IAI39] | [YP_002381778.1 , YP_002410714.1](http://www.ncbi.nlm.nih.gov/protein/418253508?report=genbank&log$=prottop&blast_rank=1&RID=FR0WJV9Y016) | 0.0 (100), 0.0 (99) |
| 46 | Antitermination Q | 34586..34948 | 120 | hypothetical protein HMPREF9428_03468 [121aa, Citrobacter freundii 4_7_47CFAA], hypothetical protein NT01EI_2298 [123aa, Edwardsiella ictaluri 93-146] | ZP_09332290.1, YP_002933704.2 | 8e-72 (88), 6e-68 (85) |
| 47 | IS1 Transposase B | 35435..35938 | 167 | IS1 transposase B [167aa, Escherichia coli str. K-12 substr. MG1655], IS1 protein InsB [167aa, Escherichia coli BL21(DE3)] | [NP_414562.1](http://www.ncbi.nlm.nih.gov/protein/16128015?report=genbank&log$=prottop&blast_rank=1&RID=J5H2N8S1013) | 1e-120 (100) |
| 48 | Unknown | complement(35932..36258) | 108 | hypothetical protein LF82_152 [168aa, Escherichia coli LF82], hypothetical protein ECEC96038_3114 [160aa, Escherichia coli EC96038] | YP_002556034.1, ZP_18653916.1 | 1e-12 (37), 2e-04 (33) |
| 49 | Unknown | complement(36284..37354) | 356 | hypothetical protein PANA5342_2839 [360aa, Pantoea ananatis LMG 5342], hypothetical protein BN132_4175 [356aa, Cronobacter turicensis 564] | YP_005196269.1, ZP_19166134.1 | 4e-161 (64), 9e-160 (63) |
| 50 | Holin | 37716..38042 | 108 | lambda family protein phage holin [108aa, Escherichia coli E1167], Holin [108aa, Escherichia fergusonii ATCC 35469] | ZP_16818293.1, YP_002381781.1 | 5e-70 (100), 4e-69 (99) |
| 51 | Lysin | 38046..38522 | 158 | [phage lysozyme family protein [158aa, Shigella flexneri 6603-63]; lysozyme [158aa, Escherichia coli RN587/1]](file:///C:\Users\u4585826.000\Desktop\SfIV-1%20final.xlsx#RANGE!alnHdr_418253514) | [ZP_12878539.1 , ZP_11522382.1](http://www.ncbi.nlm.nih.gov/protein/418253514?report=genbank&log$=prottop&blast_rank=1&RID=FCF3S9XE01R) | 4E-113 (100) , 2e-111 (99) |
| 52 | Rz (i-spanin) | 38506..38898 | 130 | putative lytic protein [130aa, Shigella flexneri 6603-63], conserved hypothetical protein [130aa, Shigella sp. D9] | [ZP_12878540.1, ZP_08393211.1](http://www.ncbi.nlm.nih.gov/protein/418253515?report=genbank&log$=prottop&blast_rank=1&RID=FCJUDW3A015) | 7e-90 (100), 4e-78 (93) |
| 53 | Rz1 (o-spanin) | 38717..39058 | 113 | putative Rz1 lytic protein [112aa, Enterobacteria phage SfV], putative Rz1 lytic protein from bacteriophage origin [107aa, Escherichia coli UMN026] | NP_599084.1, YP_002411639.1 | 5e-57 (88), 2e-56 (87) |
| 54 | Unknown | 39350..39700 | 116 | HNH endonuclease family protein [116aa, Shigella flexneri 6603-63]; endonuclease HnhC [116aa, Escherichia coli UMNK88] | [ZP_12878541.1, YP_006132114.1](http://www.ncbi.nlm.nih.gov/protein/418253516?report=genbank&log$=prottop&blast_rank=1&RID=FEDHHBH701R) | 3E-79 (100), 3e-77(98) |
