# Supplementary figures and images for: Isolation, characterization and comparative genomics of bacteriophage SfIV: a novel serotype converting phage from Shigella flexneri
Source: BMC Genomics. 2013 Oct 3;14:677. doi: 10.1186/1471-2164-14-677 (PMC3851460; doi:10.1186/1471-2164-14-677)

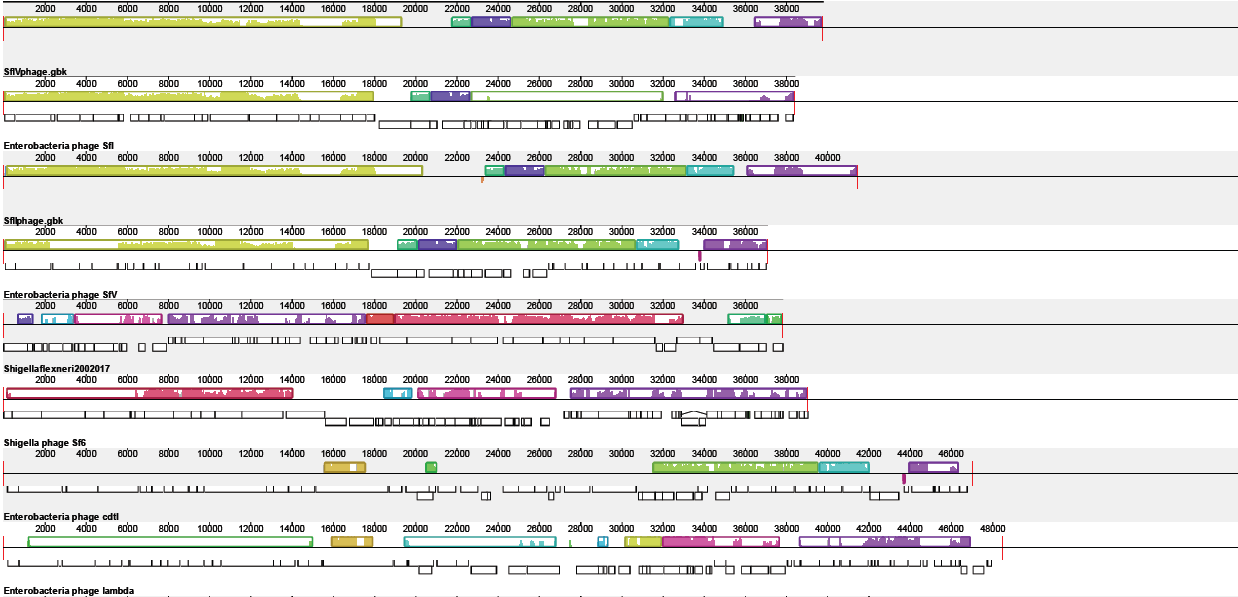


**
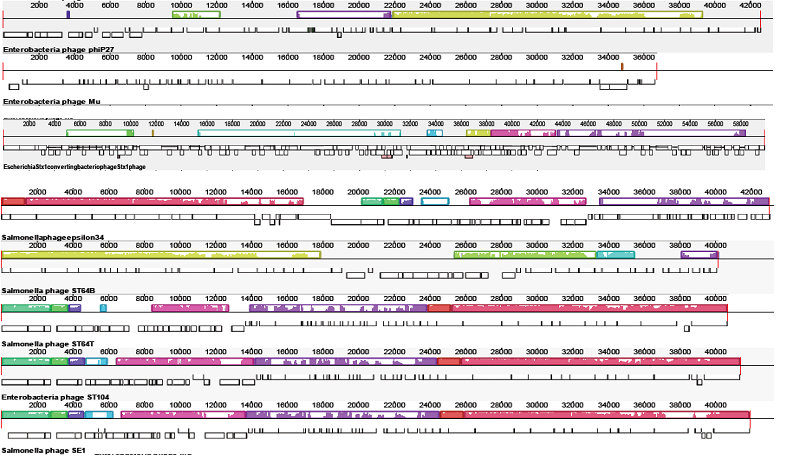
**

**­Figure S1: DNA sequence alignment of SfIV phage with other related phages**

Supplement: Additional file 2: Figure S1 — DNA sequence alignment of SfIV phage with other related phages. The alignment of SfIV phage with other phages from S. flexneri, E. coli and Salmonella. The results obtained using Progressive Mauve with default parameters showing high degree of similarity of SfIV with S. flexneri phage SfII, SfV and SfI. Coloured outlined blocks surround regions of the genome that aligned to part of another genome. The degree of DNA sequence similarity is indicated by the height of the coloured bars inside the blocks. [file 1471-2164-14-677-S2.docx]
